# Reduced Functional Connectivity in Nucleus Accumbens Subregions Associates With Cognitive Changes in Alzheimer's Disease

**DOI:** 10.1002/brb3.70440

**Published:** 2025-03-26

**Authors:** Lefan Yu, Mengmeng Feng, Yi Shang, Zhaohai Ren, Hanqi Xing, Yue Chang, Ke Dong, Yao Xiao, Hui Dai

**Affiliations:** ^1^ Department of Radiology the First Affiliated Hospital of Soochow University Suzhou People's Republic of China; ^2^ Institute of Medical Imaging Soochow University Suzhou People's Republic of China

**Keywords:** Alzheimer's disease, nucleus accumbens, resting‐state fMRI, reward circuit, subregion

## Abstract

**Background and Purpose:**

The nucleus accumbens (NAc), an important component of the reward circuit, is believed to play an indispensable role in Alzheimer's disease (AD). This study aimed to explore alterations in the functional connectivity (FC) of NAc subregions in AD patients and to explore their associations with neuropsychological profiles.

**Methods:**

Total 45 AD patients and 41 healthy controls (HCs) were recruited for this study. Four subregions of the NAc were used as regions of interest for whole‐brain FC analysis. Correlation analyses were conducted to explore the relationships between the changed FC of brain regions with significant differences and neuropsychological profiles.

**Results:**

Compared with HCs, decreased FC was observed between NAc subregions and regions of the orbitofrontal cortex (OFC), precuneus (PCUN), insula (INS), cerebellum 8, and putamen in AD patients (Gaussian random field [GRF] corrected, voxel‐level *p* < 0.001, cluster‐level *p* < 0.05). Furthermore, the FC between the left core and left PCUN was correlated with the score of the auditory verbal learning test immediate recall task in AD patients (*r* = 0.441, *p* = 0.003, Bonferroni corrected).

**Conclusion:**

Disruptions in connectivity between the NAc subregions and important cognitive‐related areas may be related to the cognitive deficits observed in AD patients, especially episodic memory function.

## Graphical abstract

This systematic literature review explores the influence of six obesity‐associated genes on multiple sclerosis (MS) risk and progression, revealing significant genetic underpinnings and potential pathways for MS pathogenesis and treatment.

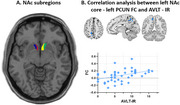

## 1 Introduction

As a common neurodegenerative disease that occurs in old and early old life (Schott et al. 2006), Alzheimer's disease (AD) is characterized by progressive cognitive decline and behavioral disorders. Clinical manifestations include loss of memory and decreased executive functions (Dubois et al. 2016; Lazarov and Hollands 2016), as well as changes in mood and personality, which seriously affect the quality of daily life (Scheltens et al. 2021). Dysfunction of reward processing is also a salient feature of AD, manifested by decreased appetite, decreased frequency of sweets, aversion to environmental sounds, and decreased tendency to gamble, which may predict increased sensitivity to satiety and sensory phenomena (Chokesuwattanaskul et al. 2023). Impaired reward processing has important effects on socioaffective dysfunction in neurodegenerative diseases.

The reward circuit is the dopaminergic (DA) mesolimbic pathway, that originates from the ventral tegmental area (VTA), and mainly connects the orbitofrontal cortex (OFC), anterior cingulate gyrus (ACC), nucleus accumbens (NAc) of the ventral striatum (VS), amygdala, and hippocampus (Dunlop and Nemeroff 2007). Each region of the participating reward circuit has particular DA activity, and its transmission defects are associated with movement (motor inability, stiffness, and static tremor), cognitive impairment (attention deficits and learning disturbances), and apathy (reduced response to stimuli related to emotion and depression) (Lauretani et al. 2022). Loss of neurons in the VTA may induce a circuit in which loss of reward with age predisposes individuals to cognitive impairment and depression, which in turn predisposes them to reactive emotional loss, increasing the likelihood of transition to dementia in older people with initial and mild cognitive impairment (Lauretani et al. 2022). Atrophy of the OFC, VS, and insula has been shown to be associated with the apathy of emotion and social reward learning disabilities (Wong et al. 2023). In addition, AD individuals have lower inter‐ and intranetwork functional connectivity (FC) in both the reward network and auditory network, suggesting that patients' access to value through reward and cultural adaptation associations is impaired (Wang et al. 2020).

As a component of the limbic system, the NAc is located in the ventromedial striatum and is considered to be an indispensable part of the reward circuit (L. Chen et al. 2022). According to its structure and function, the NAc can be divided into two parts: the core subregion and the shell subregion. The core subregion has obvious connections with the mediolateral OFC, ACC, and subcortical structures such as the thalamus, amygdala, and substantia nigra and is related to instrumental learning and behaviors that achieve motivation‐related goals. In contrast, the shell subregion has significant connections with the ventral medial prefrontal cortex (vmPFC), subcallosal cortex, regions of the mesolimbic system (VTA and hippocampus), and other subcortical structures and is involved in the integration of motivational value and novelty to promote the avoidance of fewer or non‐rewarding stimuli. These stimuli may hinder optimal reward prediction stimuli (Xia et al. 2017).

A reduced number of cholinergic neurons has been observed in the VS of patients with AD in previous studies (Lehéricy et al. 1989). A previous study revealed that apathy and loss of will exist in patients with subcortical structural pathological changes, including the NAc (Phillips et al. 1987). This indicates that the loss of cholinergic neurons in the NAc may lead to a series of behavioral disorders. Moreover, later studies on animal models of AD (Tg2576 mice) showed reduced DA projections from the VTA to the NAc (Bayassi‐Jakowicka et al. 2022), and glutamatergic transmission from the ventral region of the hippocampus to the NAc core was disrupted (A. Chen et al. 2021), resulting in dysregulated interactions among the VTA, hippocampus, and NAc. These changes occur even before the formation of amyloid plaques. Recently, studies of deep brain stimulation (DBS) promoting brain memory function have focused on the NAc. In DBS studies in AD patients, the sum score of their clinical dementia rating scales indicated a slower rate of decline when DBS targeted the VS (Picton et al. 2023).

Previous studies on the NAc in AD patients have focused mostly on morphological analysis. Some studies have shown that disease progression is associated with damage to the mesolimbic system (including bilateral atrophy of the NAc and hippocampus) (Carlesimo et al. 2015; Topkan et al. 2022). Other studies have indicated that the volume changes in the NAc are manifested in different subregions, while the overall volume changes are not significant (Nie et al. 2017). To date, very few resting‐state functional magnetic resonance imaging (rs‐fMRI) studies have focused on the relationship between the NAc and AD. These studies found that the average brain activity and regional spontaneous activity were reduced in the NAc in AD patients than in normal controls (Kazemifar et al. 2017; Lin et al. 2022). In addition, higher inflammatory cytokine marker levels in AD patients were associated with lower FC between the NAc and regions involved in memory, visual, and language processing. And the APOE4 allele moderated the relationship (Contreras et al. 2020). Although previous neuroimaging studies have shown that alterations in the volume of the NAc are associated with AD development, the effects of FC changes in NAc subregions have not yet been fully elucidated.

In this study, rs‐fMRI was conducted to explore FC changes in subregions of the NAc in AD patients. In addition, this study aimed to examine the relationships between these changes and neuropsychological profiles.

## 2 Materials and Methods

### 2.1 Subjects

Fifty AD patients and 44 healthy controls (HCs) were included in this study, and the study received approval from the Ethics Committee of The First Affiliated Hospital of Soochow University. All of the subjects were recruited from the Geriatric Department of the First Affiliated Hospital of Soochow University in 2018–2022 and underwent a series of comprehensive neuropsychological tests after written informed consents proceeded. The general cognition of all the subjects was evaluated by using Montreal Cognitive Assessment (MoCA) (Nasreddine et al. 2005) and Mini‐Mental State Examination (MMSE) (Lu et al. 2011). To evaluate depression severity, the Hamilton Rating Scale for Depression (HAMD) (Hamilton 1960) was used; to evaluate anxiety levels, the Hamilton Anxiety Rating Scale (HAMA) (Hamilton 1959) was used. The auditory verbal learning test (AVLT), including the immediate recall task and delayed recall task, was conducted to measure episodic memory function (Zhao et al. 2012). Executive function was evaluated by the Stroop color‐word test, which includes measurements of speed (Task A) and accuracy (Task B) (Houx et al. 1993). The Clock‐Drawing Test (CDT) (Wiechmann et al. 2010), the verbal fluency test (VFT) (Tombaugh et al. 1999), and the digital span test (DST) (Leung et al. 2011) were used to assess visuospatial function, language function, and attention function, respectively.

The diagnosis of AD was referred to the National Institute on Aging‐Alzheimer's Association criteria (McKhann et al. 2011). In addition, the recruited AD patients also needed to satisfy the inclusion criteria as follows: (1) with a MOCA score ≤ 18; and (2) with a white matter hyperintensities (WMH) score less than or equal to 2, which was assessed according to the Fazekas criteria by fluid attenuated inversion recovery (FLAIR) images (Supporting Information). The inclusion criteria for the HCs included: (1) age and sex‐matched healthy volunteers who had no cognitive complaints; (2) with a MOCA score ≥ 26; and (3) no structural abnormality found on conventional MR imaging. The exclusion criteria for all the subjects were as follows: (A) had evidence of obvious central nervous system diseases; (B) had a history of psychiatric or metabolic disorders; and (C) had alcohol or drug abuse.

### 2.2 MRI Data Acquisition

Our MRI data were acquired on a GE Signa HDxt 3.0 T MR scanner using an eight‐channel head coil. All the subjects were asked to keep their heads and limbs still, eyes closed, and not to fall asleep. The 3D T1‐weighted structural images were obtained with the following parameters: repetition time (TR) = 6.50 ms, echo time (TE) = 2.8 ms, slice thickness/gap = 1/0 mm, flip angle = 7°, field of view (FOV) = 256 × 256 mm^2^, and 176 sagittal slices. Resting‐state blood oxygen level‐dependent images were acquired using gradient‐echo echo‐planar sequences with the following parameters: TR = 2000 ms, TE = 30 ms, slice thickness/gap = 4/0 mm, flip angle = 90°, FOV = 256 × 256 mm^2^, image matrix size = 64 × 64, 33 axial slices, and the scanning time was 8 min. T2‐weighted FLAIR images were obtained with the following parameters: TR = 8000 ms, TE = 147 ms, slice thickness/gap = 5/1.5 mm, FOV = 240 × 240 mm^2^, image matrix size = 256 × 192, and the sequences were used to measure WMH.

### 2.3 Image Processing

The data were processed via Data Processing and Analysis for Brain Imaging (DPABI version 6.1, http://rfmri.org/DPABI) (Yan et al. 2016) toolboxes on the Matlab R2021b (MathWorks, Natick, Massachusetts, USA). All images were transformed from Digital Imaging and Communications in Medicine (DICOM) format to Neuroimaging Informatics Technology Initiative (NIFTI) format file. The main preprocessing steps of the rs‐fMRI data were as follows: removing the first 10 time points to eliminate image noise generated before the gradient magnetic field was stable; slice timing correction was conducted on the obtained 230 time points before head motion correction was carried out; co‐registration of the structural image to the functional image; structural image segmentation by Diffeomorphic Anatomical Registration Through Exponentiated Lie Algebra (DARTEL); polynomials were used to eliminate linear trends and nuisance covariates regression, including white matter signal, cerebrospinal fluid signal, and 24‐Friston head motion parameters; the DARTEL method was used to generate the group template, and the template was normalized into the standard Montreal Neurological Institute (MNI) brain template, resampled to 3 × 3 × 3 mm^3^ voxels; spatial smoothing was performed on the derived volumes with a Gaussian smoothing kernel of 4 mm full width at half maximum (FWHM), which is in accordance with previous studies on the NAc subregions (L. Chen et al. 2022; Hu et al. 2023); and a temporal filter with 0.01–0.08 Hz. The inclusion threshold for head motion was defined as head translation < 3 mm or rotation < 3°, and the mean framewise displacement (FD) parameters were evaluated to control head motion.

### 2.4 Regions of Interest (ROIs) and Functional Connectivity Analysis

**FIGURE 1:**
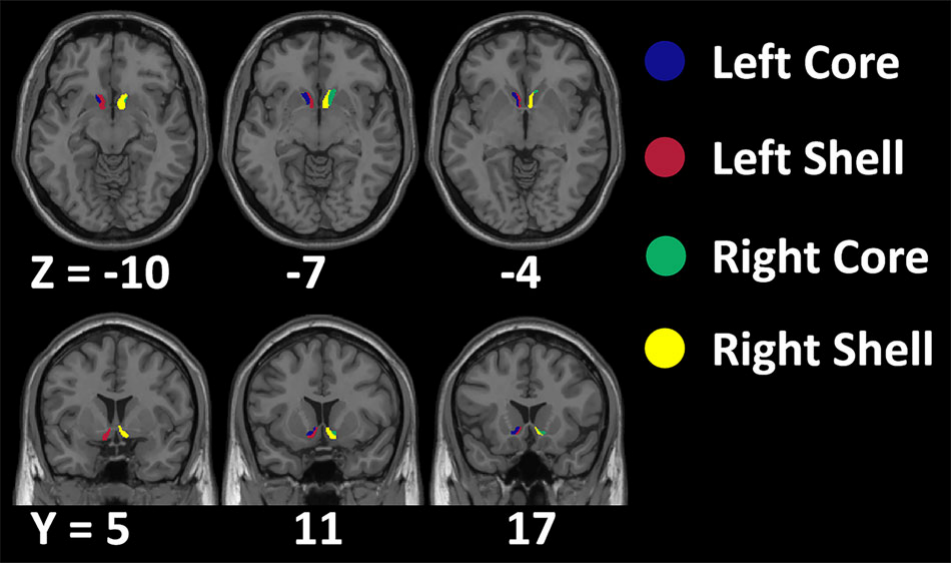
The four NAc subregions characterized by Cartmell et al. (2019).

For the regions of interest (ROIs), the bilateral NAc core subregions and NAc shell subregions were chosen for further analysis (Figure 1). The core subregion and shell subregion were delimited according to a NAc subregion probabilistic atlas (Cartmell et al. 2019), which has more minor nondeterminacy derived during the extraction and processing stream than previous study (Xia et al. 2017) due to the large amount of participants in their dataset.

ROI‐based FC was calculated via DPABI software. The reference time courses of each ROI were acquired by numerating the mean time courses of all of the voxels within each ROI. The correlation between the ROI reference time courses and time courses of the whole brain was determined by performing a Pearson correlation at a voxel level. When transformed into a z‐map, the normality of the correlation coefficient map was improved by utilizing Fisher's r‐to‐z transformation. The DPABI software was also used to compare volume differences between the four subregions by extracting the mean gray matter volume.

To validate the exactitude of the subregion partition in the NAc, one‐sample *t* tests were conducted for the FC‐map of the HC group and the AD group to recognize regions presenting significant connectivity with all of the subregions of NAc within the two groups (Gaussian random field (GRF) corrected, voxel‐wise *p* < 0.001; cluster‐level *p* < 0.05). The names of the regions with significant differences were derived from the automated anatomical labeling atlas (Rolls et al. 2020). Differences in FC between the ipsilateral subregions of the NAc in the HC group were examined by carrying out paired‐samples *t* tests (GRF corrected, voxel‐wise *p* < 0.001; cluster‐level *p* < 0.05).

### 2.5 Statistical Analysis

IBM SPSS Statistics 20 software (Chicago, IL, USA) was used for statistical analysis of demographic and neuropsychological data. Distribution normality was checked by the Kolmogorov–Smirnov test. The differences in demographic information, neuropsychological test scores, and mean volume between the AD group and the HC group were compared by performing two‐sample *t* tests, Mann–Whitney *U* tests, and chi‐square (*χ* ^2^) tests. With age, gender, and years of education as covariates, for the analysis of FC, two‐sample *t* tests were carried out to compare the FC differences between the AD group and the HC group across all NAc subregions (GRF corrected, voxel‐wise *p* < 0.001; cluster‐level *p* < 0.05). The extent threshold was 20 voxels. In addition, the correlations between the connectivity strength acquired from the significant regions and the neuropsychological scales in AD group were investigated by using partial correlation analysis, with age, gender, and years of education as covariates (*p* < 0.05). The Bonferroni method was used for multiple comparison correction.

In addition, we calculated the power for *t* tests of the NAc subregions between the AD and HC groups by using G Power Software (version 3.1.9.7). The details are described in the Supporting Information.

## 3 Results

### 3.1 Demographic and Neuropsychological Data

Five subjects from the AD group and 3 subjects from the HC group were excluded due to head motion. As presented in Table 1, there were no significant differences in the age, gender, years of education, mean volume of NAc subregions, and scores on the HAMA and the HAMD (all *p* > 0.05) of the subjects in the AD and HC groups. Other neuropsychological test scores including episodic memory function, visuospatial function, language function, attention function, and executive function were significantly different between the two groups (all *p* < 0.001).

**TABLE 1:** Demographic and clinical features of participants.

Characteristics	AD (*n* = 45)	HC (*n* = 41)	*t* value	*p* value
Gender (M/F)	13/32	20/21	3.590	0.058^a^
Age (years)	68.78 ± 7.87	69.95 ± 8.01	−0.685	0.495
Education (years)	8.87 ± 4.91	10.76 ± 3.38	−1.513	0.130
Left core (cm^3^)	0.45 ± 0.27	0.51 ± 0.24	−1.148	0.254
Left shell (cm^3^)	0.46 ± 0.25	0.51 ± 0.23	−1.081	0.283
Right core (cm^3^)	0.44 ± 0.25	0.51 ± 0.24	−1.476	0.144
Right shell (cm^3^)	0.42 ± 0.22	0.49 ± 0.22	−1.577	0.119
MoCA	12.69 ± 3.93	27.44 ± 1.78	−7.998	0.000^*^
MMSE	16.04 ± 5.30	28.12 ± 1.40	−7.980	0.000^*^
HAMA	2.91 ± 2.76	2.90 ± 4.00	−0.931	0.352
HAMD	3.31 ± 4.30	2.66 ± 4.39	−1.311	0.190
AVLT‐IR	6.91 ± 4.31	19.07 ± 4.19	−13.241	0.000^*^
AVLT‐DR	0.56 ± 1.44	6.15 ± 2.68	−7.526	0.000^*^
Stroop task A(s)	32.09 ± 25.29	16.45 ± 9.81	3.844	0.000^*^
Stroop task B	3.07 ± 4.23	0.68 ± 1.85	−3.569	0.000^*^
VFT	9.29 ± 4.04	16.68 ± 4.15	−8.365	0.000^*^
CDT	1.47 ± 0.99	3.93 ± 0.26	−7.920	0.000^*^
DST	9.31 ± 2.07	12.68 ± 1.77	−8.097	0.000^*^

Abbreviations: AVLT‐DR, auditory verbal learning test delayed recall; AVLT‐IR, auditory verbal learning test immediate recall; CDT, Clock‐Drawing Test; DST, digital span test; HAMA, Hamilton Anxiety Rating Scale; HAMD, Hamilton Rating Scale for Depression; MMSE, Mini‐Mental State Examination; MoCA, Montreal Cognitive Assessment; VFT, verbal fluency test.

^a^chi‐square test.

*p < 0.01.

### 3.2 Differences in FC and Correlational Analysis

**FIGURE 2:**
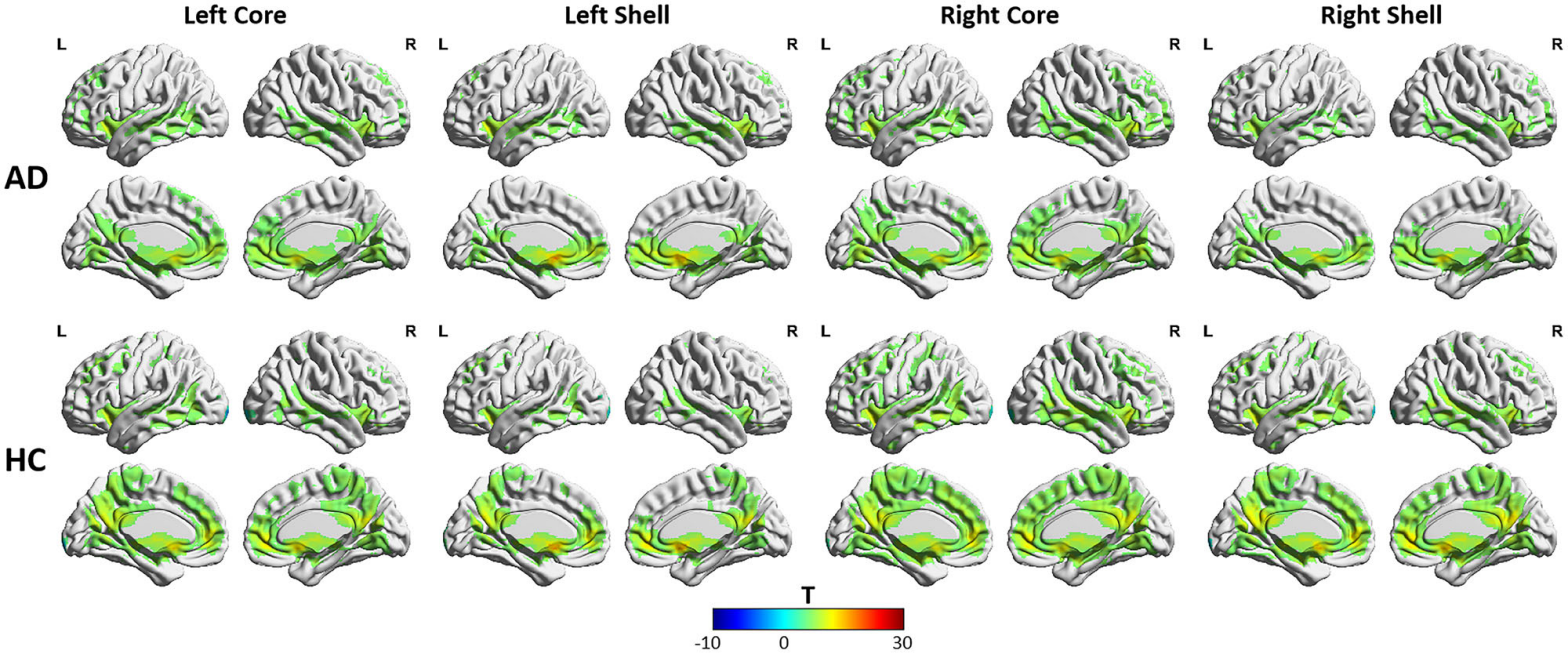
Spatial distributions of the FC of the four NAc subregions within the AD group and the HC group. FC data were projected onto a surface brain using BrainNet Viewer (https://www.nitrc.org/projects/bnv/). The color bar scale represents *t* values.

**FIGURE 3:**
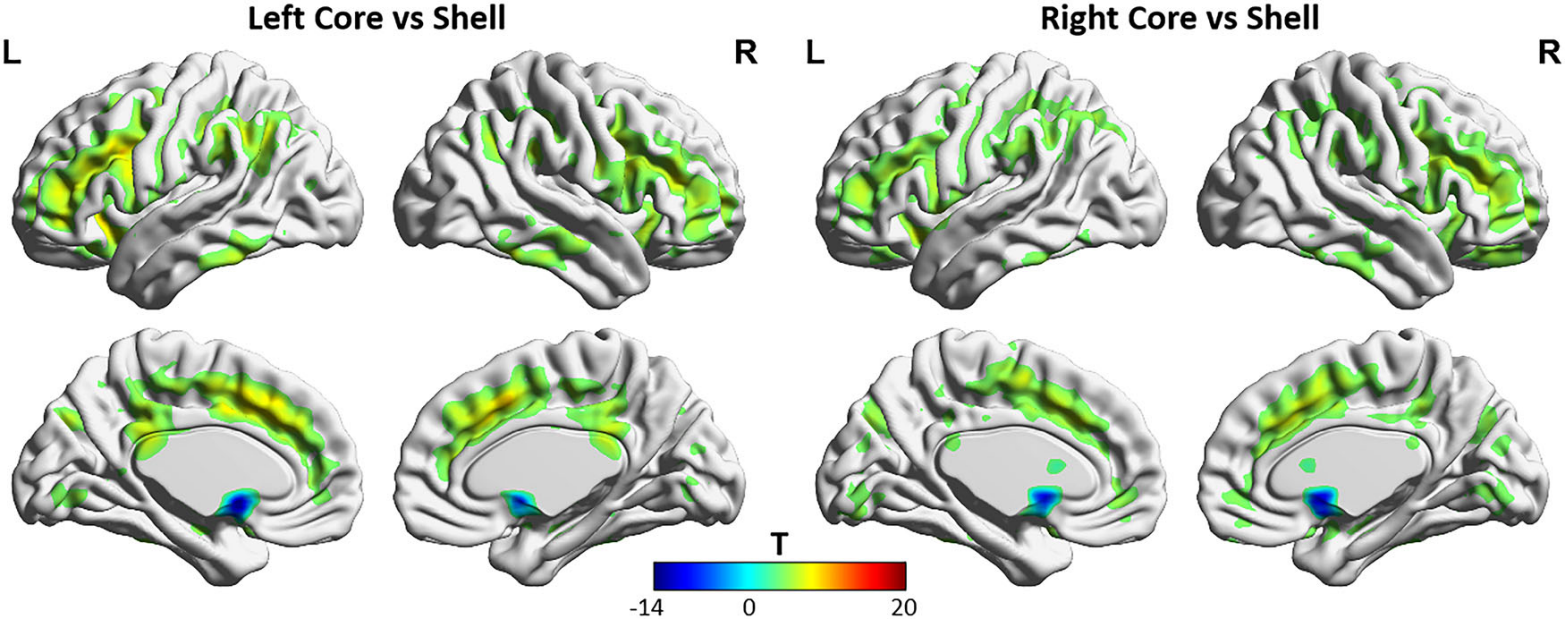
Regions showing significant differences in rsFC with the NAc core subregion compared with the NAc shell subregion across the participants. Warm colors represent the regions showing greater FC with the NAc core subregion compared with the NAc shell subregion. Cool colors represent the regions showing greater FC with the NAc shell subregion compared with the NAc core subregion. The images were created using BrainNet Viewer (https://www.nitrc.org/projects/bnv/). The color bar scale represents *t* values.

All of the subregions of the NAc were positively connected to regions of the frontal lobe, temporal lobe, ACC, basal ganglia, subcallosal gyrus, OFC, thalamus, and hippocampus in both the AD group and the HC group, according to one‐sample *t* test analyses (Figure 2) (GRF corrected, voxel‐wise *p* < 0.001; cluster‐level *p* < 0.05). The NAc core subregions presented stronger FC with the regions of the frontal lobe and ACC compared with the shell subregions, as well as weaker FC with the subcallosal gyrus, according to paired sample *t* test analyses (GRF corrected, voxel‐wise *p* < 0.001; cluster‐level *p* < 0.05). The differences observed in FC within the NAc core subregion and shell subregion were in accordance with previous studies (Cartmell et al. 2019; Xia et al. 2017) (Figure 3).

**FIGURE 4:**
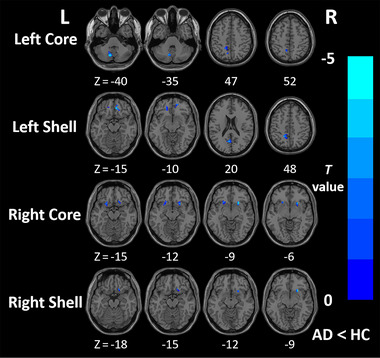
Results of functional connectivity. Brain regions with reduced functional connectivity with the left core, left shell, right core, and right shell of NAc in AD patients compared with HC. The statistical threshold was set at *p* < 0.001 with a cluster‐level of *p* < 0.05 (two‐tailed, GRF corrected), and the extent threshold was 20 voxels.

**FIGURE 5:**
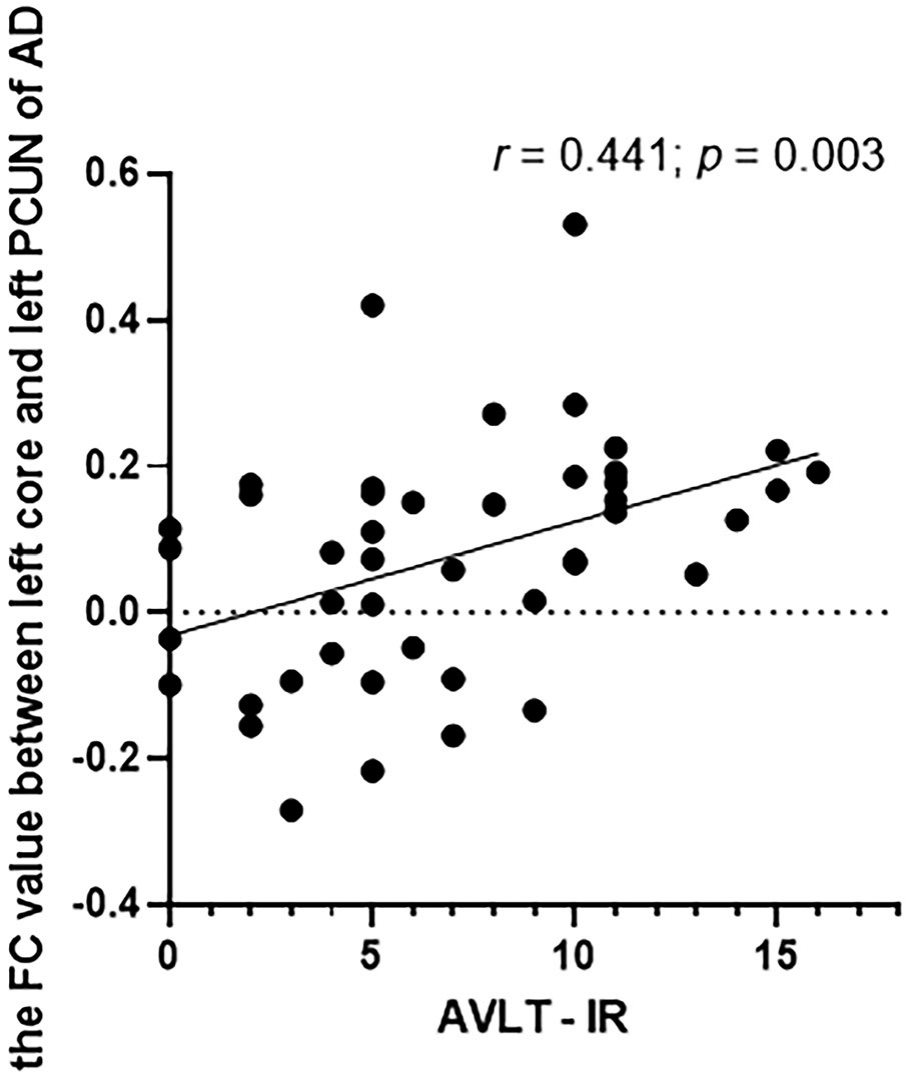
The correlation between AVLT‐IR and the FC value between left core and left PCUN in AD. AD, Alzheimer disease; AVLT‐IR, auditory verbal learning test immediate recall; FC, functional connectivity; PCUN, precuneus.

The differences in FC between the AD group and the HC group in the four NAc subregions are presented in Figure 4 and Table 2 (GRF corrected, voxel‐wise *p* < 0.001; cluster‐level *p* < 0.05). The left core and left shell commonly showed decreased FC with the left precuneus (PCUN) (both *p* < 0.001). The right core and right shell showed decreased FC with the right putamen (PUT) (both *p* < 0.001). In addition, the left core showed decreased FC with lobule VIII of the left cerebellar hemisphere (CER8) (*p* < 0.001) while the left shell showed decreased FC with the right superior frontal gyrus, medial orbital (PFCventmed) (*p* < 0.001), left medial orbital gyrus (OFCmed) (*p* < 0.001), and left cuneus (CUN) (*p* = 0.001). In addition, the right core also showed decreased FC with the left insula (INS) (*p* < 0.001). The reduced FC between the left core and the left PCUN was associated with the score of CDT (*r* = 0.319, *p* = 0.039). The reduced FC between the right core and the left INS was associated with the score of HAMA scale (*r* = −0.310, *p* = 0.046). Furthermore, the reduced FC between the left PCUN and the left NAc subregions was associated with the score of AVLT immediate recall task (*r* = 0.441, *p* = 0.003; *r* = 0.330, *p* = 0.033). However, after the Bonferroni correction, only the FC between left PCUN and left core survived (Table S1 and Figure 5).

**TABLE 2:** The significant between‐group differences in functional connectivity for the NAc subregions.

Seeds	Brain region	Brodmann area	Peak MNI coordinate			Peak *t* value	Cluster voxels
			X	Y	Z		
Left core	Left CER8	—	−12	−69	−39	−4.6723	28
	Left PCUN	BA5L	−12	−54	57	−3.9369	31
Left shell	Right PFCventmed	BA11R	9	39	−15	−4.7361	37
	Left OFCmed	BA11L	−18	42	−22	−4.1677	26
	Left CUN	BA23L	−7	−64	21	−4.3391	21
	Left PCUN	—	−13	−48	48	−4.2544	23
Right core	Left INS	BA48L	−25	17	−15	−4.3858	35
	Right PUT	BA11R	24	18	−19	−4.9875	34
Right shell	Right PUT	BA11R	24	18	−19	−4.5066	22

Abbreviations: CER8, lobule VIII of left cerebellar hemisphere; CUN, cuneus; INS, insula; MNI, Montreal Neurological Institute; OFCmed, medial orbital gyrus; PCUN, precuneus; PFCventmed, superior frontal gyrus, medial orbital; PUT, putamen.

## 4 Discussion

In this study, we investigated FC changes of NAc subregions in AD by using rs‐fMRI and explored their associations with neuropsychological profiles. Our results found that the FC between the four subregions of the NAc and different brain regions, including the OFC/Brodmann area 11, PCUN, CUN, INS, PUT, and CER8, was reduced. In addition, reduced FC between the left core and the left PCUN was associated with poorer episodic memory function.

In previous rs‐fMRI studies, average brain activity and regional spontaneous activity changes in the NAc, PUT, hippocampus, and INS were observed in the AD group (Kazemifar et al. 2017; Lin et al. 2022). Kazemifar et al. (2017) reported that average brain activity, especially in the NAc, is significantly lower in AD patients than in HCs. These results underscore the specificity of NAc function changes in AD, and our study highlights FC changes in the NAc at the subregion level. However, the FC changes between the hippocampus and NAc were not observed in this study. Hippocampal lesions are known to play an important role in the cognitive decline of AD patients, and the NAc‐hippocampus circuit is also recognized as an important pathway for NAc involvement in memory encoding (A. Chen et al. 2021). AD patients may maintain FC between the NAc and the hippocampus through compensatory mechanisms of enhanced connectivity in other regions, which requires further investigation.

In this study, reduced FC was found between the left NAc and left PCUN in AD patients and was positively correlated with the performance on the AVLT immediate recall task. In the early stages of AD, neuropathological alterations are distributed mainly in the posterior cortex of the brain (Haupt et al. 2008). The PCUN is not only the most salient region of tau pathological deposition and neuroinflammation (Yokoi et al. 2018), but also the main part of the default mode network (DMN). The DMN is closely associated with human brain functions such as monitoring the internal and external environment, emotion processing, self‐introspection, and episodic memory extraction (Benhamou et al. 2021). In a previous study, AD patients showed decreased gray matter volume in the PCUN, accompanied by abnormal activation and decreased FC in memory tasks (Gili et al. 2011). Cui et al. (2022) also reported that repetitive transcranial magnetic stimulation could improve verbal memory function in older adults by increasing FC in the bilateral PCUN within the DMN. Notably, some researchers have reported that computerized cognitive training can improve language memory function by downregulating the FC between the PCUN and the posterior cingulate gyrus in older adults (Wu et al. 2023). However, this does not conflict with our research results. On the one hand, the subjects in the study by Wu et al. were selected from patients with mild cognitive impairment. They suggested that computerized cognitive training reversed the compensatory mechanisms that elderly individuals use to maintain cognitive function (Wu et al. 2023), whereas the compensatory function in patients with AD may have already been damaged. On the other hand, the brain regions connected to the PCUN also differed between the two studies. The DA pathway can enhance episodic memory function by regulating memory‐related brain regions. In summary, we infer that the NAc may be involved in the processing of episodic memory function through connections to the PCUN (Shigemune et al. 2014). The NAc core and the PCUN may be vulnerable areas for impaired episodic memory in AD patients.

The role of the DMN in emotion processing is also crucial (Benhamou et al. 2021). However, the strength of FC between the NAc and PCUN and the score of mood scale were not correlated in this study. The possible reason is that subjects in the AD group scored low on both scales, belonging to a state without anxiety or depression, and therefore, a correlation between FC and mood could not be observed. Future studies with or without information on neuropsychiatric symptoms and the NAc subregion are warranted. On the other hand, the DMN can be distinguished into core and posterior parts that are responsible for theory of mind and episodic memory (Dadario and Sughrue 2023). The results presented in this study show a relatively broader distribution across the posterior regions.

Reduced FC between the NAc shell and the OFC was observed in this study. The mesolimbic and mesocortical pathways, two different DA pathways, mainly project to regions of the NAc and OFC, which have long been considered to play a main role in reward processing (Huckins et al. 2019). In the intertemporal decision‐making process, AD patients present reduced delayed reward selection (Geng et al. 2020). One study reported that AD patients were unable to establish relationships between stimuli and rewards (Alameda‐Bailen et al. 2017). When alternative choices are faced, the features of each choice are transformed into value‐based information, and the NAc and OFC are closely related to the representation of these information values (Liu et al. 2011). The NAc shell (but not the core subregion) can reduce the preference for less rewarded or more uncertain choices when processing multiple choices, thus inhibiting unrewarded or unrelated behaviors (Floresco 2015).

In the AD groups, the FC between the left core and posterior cerebellum was decreased. The cerebellum seems to integrate multiple modes of function, in addition to maintaining balance and participating in motor coordination, involving learning and memory, emotional processing, attention, and other functions (Yue et al. 2023). Reduced FC was also found between the right core and left INS in AD patients. The INS is involved in the regulation of autonomous and physiological responses to reward and emotional stimuli (Hu et al. 2023). Furthermore, the right NAc core presented reduced FC with the right PUT. Extensive intrastriatal projection connections between the NAc core and the PUT (van Dongen et al. 2005), as well as more pronounced resting‐state FC (Xia et al. 2017) than the shell subregion, have been reported in previous studies of subcortical volume in AD patients. Therefore, the demarcation between the NAc core and the PUT may not be as clear due to the large contact area. Moreover, the results of this study could reveal bilateral interhemispheric differences in FC changes in the NAc. FC changes were observed between the left NAc and the OFC and between the right NAc and the INS, which is consistent with the findings of previous studies (Cartmell et al. 2019).

This study had several limitations. First, the sample size was relatively small, and further studies with larger samples are needed to confirm its reproducibility. Second, we assessed the subjects' different cognitive domains via clinical cognitive examinations, and the results might not intuitively reflect changes in the subjects' reward processing. Future studies are encouraged to employ more comprehensive reward‐related event‐related fMRI to assess reward‐related impairments more comprehensively. In addition, this was a cross‐sectional study, and longitudinal studies are required to explore neuropsychological profile changes in AD and their relationship with the development of associated clinical symptoms in the future.

In conclusion, this study found that FC was significantly reduced in the four subregions of the NAc in AD patients. These reductions were associated with various brain regions linked to cognition, decision‐making, and emotional functions. The observed correlation between changes in FC and cognitive performance highlights the importance of these brain regions in cognitive processes.

## Author Contributions

**Lefan Yu**: data curation, writing – original draft, formal analysis. **Mengmeng Feng**: investigation. **Yi Shang**: investigation. **Zhaohai Ren**: investigation. **Hanqi Xing**: data curation, methodology. **Yue Chang**: methodology, data curation. **Ke Dong**: methodology, data curation. **Yao Xiao**: methodology, data curation. **Hui Dai**: conceptualization, writing – review and editing.

## Ethics Statement

The study received approval from the Ethics Committee of The First Affiliated Hospital of Soochow University (number 2024 the 317th). All participants provided written informed consent.

## Conflicts of Interest

The authors declare no conflicts of interest.

### Peer Review

The peer review history for this article is available at https://publons.com/publon/10.1002/brb3.70440.

## Supporting information



Supporting Information

## Data Availability

The MRI data will be made available on reasonable request.
