# Supplementary material for: Reduced Functional Connectivity in Nucleus Accumbens Subregions Associates With Cognitive Changes in Alzheimer's Disease
Source: Brain Behav. 2025 Mar 26;15(3):e70440. doi: 10.1002/brb3.70440 (PMC11938111; doi:10.1002/brb3.70440)
Supplement: Supplementary file 1 — Supporting Information [file BRB3-15-e70440-s001.docx]

**Fazekas criteria (DOI: 10.1177/0271678X16635657)**

The scoring of T2-FLAIR images is based on the Fazekas rating scale, which evaluates both the periventricular and deep white matter hyperintensities (WMH) separately and then sums the scores from both categories to obtain a total score. The scoring criteria are as follows:

**Periventricular Scoring:**

0 points: No abnormalities;

1 point: Abnormality in the anterior horn or occipital horn, or pencil-thin periventricular hyperintensities;

2 points: Smooth halo surrounding the lateral ventricles;

3 points: Irregular periventricular hyperintensity extending into the deep matter.

**Deep Matter Scoring:**

0 points: No abnormalities;

1 point: Punctate lesions;

2 points: Patchy areas of abnormal hyperintensity with a tendency for lesions to merge or single lesions larger than 3mm in diameter

3 points: Large areas of lesions with a patchy and irregular pattern of abnormal hyperintensity.

The-quantitative assessment of WMH yields total score ranging from 0 to 6 points.

**Table S1** Correlation between FC values and neuropsychological scales in AD patients

| Variables |  | LC1 | LC2 | LS1 | LS2 | LS3 | LS4 | RC1 | RC2 | RS1 |
| --- | --- | --- | --- | --- | --- | --- | --- | --- | --- | --- |
| VFT | *R* value | 0.006 | 0.124 | 0.145 | 0.103 | 0.129 | 0.091 | 0.062 | 0.102 | 0.144 |
|  | *P* value | 0.972 | 0.434 | 0.358 | 0.518 | 0.415 | 0.567 | 0.695 | 0.521 | 0.362 |
| CDT | *R* value | 0.005 | 0.319 | 0.304 | 0.266 | -0.124 | 0.288 | 0.052 | 0.158 | 0.178 |
|  | *P* value | 0.976 | 0.039* | 0.050 | 0.088 | 0.432 | 0.064 | 0.742 | 0.316 | 0.260 |
| DST | *R* value | 0.046 | 0.163 | 0.059 | 0.025 | 0.083 | 0.127 | 0.030 | -0.114 | -0.098 |
|  | *P* value | 0.773 | 0.301 | 0.710 | 0.876 | 0.599 | 0.422 | 0.849 | 0.474 | 0.538 |
| AVLT-IR | *R* value | 0.060 | 0.441 | 0.272 | 0.211 | 0.102 | 0.330 | 0.180 | 0.157 | 0.216 |
|  | *P* value | 0.704 | 0.003** | 0.082 | 0.181 | 0.520 | 0.033* | 0.255 | 0.322 | 0.170 |
| AVLT-DR | *R* value | -0.002 | 0.254 | 0.233 | 0.175 | 0.117 | 0.119 | 0.265 | 0.144 | 0.145 |
|  | *P* value | 0.991 | 0.104 | 0.138 | 0.269 | 0.459 | 0.451 | 0.090 | 0.363 | 0.360 |
| Stroop task A | *R* value | 0.219 | 0.174 | -0.054 | -0.039 | 0.004 | 0.138 | 0.040 | 0.118 | 0.073 |
|  | *P* value | 0.164 | 0.272 | 0.735 | 0.808 | 0.981 | 0.383 | 0.800 | 0.455 | 0.646 |
| Stroop task B | *R* value | -0.051 | -0.198 | -0.148 | -0.131 | -0.159 | -0.250 | -0.141 | -0.117 | -0.232 |
|  | *P* value | 0.749 | 0.210 | 0.348 | 0.410 | 0.315 | 0.111 | 0.373 | 0.462 | 0.139 |
| HAMA | *R* value | -0.179 | -0.135 | -0.058 | -0.107 | 0.020 | -0.119 | -0.310 | -0.157 | -0.159 |
|  | *P* value | 0.258 | 0.394 | 0.717 | 0.500 | 0.902 | 0.455 | 0.046* | 0.321 | 0.314 |
| HAMD | *R* value | -0.176 | -0.079 | -0.197 | -0.229 | -0.128 | -0.037 | -0.296 | -0.167 | -0.176 |
|  | *P* value | 0.264 | 0.618 | 0.212 | 0.145 | 0.420 | 0.817 | 0.057 | 0.291 | 0.264 |

LC1 = left core and lobule VIII of left cerebellar hemisphere; LC2 = left core and left precuneus; LS1 = left shell and right superior frontal gyrus, medial orbital; LS2 = left shell and left medial orbital gyrus; LS3 = left shell and left cuneus; LS4 = left shell and left precuneus; RC1 = right core and left insula; RC2 = right core and right putamen; RS1 = right shell and right putamen; VFT = verbal fluency test; CDT = Clock-Drawing Test; DST = digital span test; AVLT-IR = auditory verbal learning test immediate recall; AVLT-DR = auditory verbal learning test delayed recall; HAMA = Hamilton Anxiety Rating Scale; HAMD = Hamilton Rating Scale for Depression

*P < 0.05

**P < 0.005 (Bonferroni corrected)

**Effect Size and Statistical Power**

We calculated the power for T-tests of the NAc subregions between the AD and HC groups by using the G Power Software (version 3.1.9.7). The method of power analysis we used was Means: Difference between two independent means (two groups). The type of power analysis was set to Post hoc. We first calculated the effect size d corresponding to our data. Then we used G * Power to calculate the power for α= 0.05. The parameter we set were as follow:

t tests - Means: Difference between two independent means (two groups)

Analysis: Post hoc: Compute achieved power

**LC-Left CER8**

Input: Tail(s) = Two

Effect size d = 1.03

α err prob = 0.05

Sample size group 1 = 45

Sample size group 2 = 41

Output: Noncentrality parameter δ = 4.7707448

Critical t = 1.9886097

Df = 84

Power (1-β err prob) = 0.9970707

**LC-Left PCUN**

Input: Tail(s) = Two

Effect size d = 0.90

α err prob = 0.05

Sample size group 1 = 45

Sample size group 2 = 41

Output: Noncentrality parameter δ = 4.1686119

Critical t = 1.9886097

Df = 84

Power (1-β err prob) = 0.9846345

**LS-Right PFCventmed**

Input: Tail(s) = Two

Effect size d = 0.88

α err prob = 0.05

Sample size group 1 = 45

Sample size group 2 = 41

Output: Noncentrality parameter δ = 4.0759761

Critical t = 1.9886097

Df = 84

Power (1-β err prob) = 0.9807265

**LS-Left OFCmed, LS-Left PCUN**

Input: Tail(s) = Two

Effect size d = 0.85

α err prob = 0.05

Sample size group 1 = 45

Sample size group 2 = 41

Output: Noncentrality parameter δ = 3.9370224

Critical t = 1.9886097

Df = 84

Power (1-β err prob) = 0.9732994

**LS-Left CUN**

Input: Tail(s) = Two

Effect size d = 0.74

α err prob = 0.05

Sample size group 1 = 45

Sample size group 2 = 41

Output: Noncentrality parameter δ = 3.4275254

Critical t = 1.9886097

Df = 84

Power (1-β err prob) = 0.9233710

**RC-Left INS**

Input: Tail(s) = Two

Effect size d = 0.87

α err prob = 0.05

Sample size group 1 = 45

Sample size group 2 = 41

Output: Noncentrality parameter δ = 4.0296582

Critical t = 1.9886097

Df = 84

Power (1-β err prob) = 0.9784745

**RC-Right PUT**

Input: Tail(s) = Two

Effect size d = 0.89

α err prob = 0.05

Sample size group 1 = 45

Sample size group 2 = 41

Output: Noncentrality parameter δ = 4.1222940

Critical t = 1.9886097

Df = 84

Power (1-β err prob) = 0.9827750

**RS-Right PUT**

Input: Tail(s) = Two

Effect size d = 0.81

α err prob = 0.05

Sample size group 1 = 45

Sample size group 2 = 41

Output: Noncentrality parameter δ = 3.7517508

Critical t = 1.9886097

Df = 84

Power (1-β err prob) = 0.9598163

Based on the results presented above, using a sample size of 86 allows us to achieve a power range 0.923-0.997 for α= 0.05.
